# Clinical characteristics and outcome of mucormycosis: A multi-center retrospective analysis in Saudi Arabia over 11 years

**DOI:** 10.1016/j.ijregi.2022.07.004

**Published:** 2022-07-08

**Authors:** Reem Abanamy, Abdulrahman Alsaud, Rawan Alabdulali, Mohammed Alsobaie, Bassam Alalwan, Sameera Aljohani, Saeed Alshieban, Hanan Turkistani, Abdullah Almohaizeie, Mohammad Bosaeed, Fahad AlRabiah

**Affiliations:** 1 Division of Infectious Diseases, Department of Medicine, King Abdulaziz Medical City-Riyadh, Saudi Arabia; 2 Department of Pathology, King Abdulaziz Medical City-Riyadh, Saudi Arabia; 3 King Saud bin Abdulaziz University for Health Sciences-Riyadh, Saudi Arabia; 4 King Abdullah International Medical Research Center-Riyadh, Saudi Arabia; 5 Division of Infectious Diseases, Department of Medicine, King Faisal Specialist Hospital and Research Center-Riyadh, Saudi Arabia; 6 Pharmaceutical Care Division, King Faisal Specialist Hospital & Research Center-Riyadh, Saudi Arabia; 7 Division of Infectious Diseases, King Abdullah Medical City-Makkah, Saudi Arabia

**Keywords:** zygomycosis, zygomycete, mucormycosis, fungal, epidemiology, Saudi Arabia

## Abstract

**Background:**

Mucormycosis is a life-threatening fungal infection with variable epidemiology between countries. Limited data are available locally; we aim to describe the clinical spectrum and outcome of mucormycosis in Saudi Arabia.

**Methods:**

A retrospective multi-center study including all patients with clinical and pathological evidence of mucormycosis in 3 tertiary care centers in Saudi Arabia from January 2009 to December 2019.

**Results:**

Thirty-three patients were identified during the study period. The mean age was 42 years. People with diabetes accounted for 48% of the patient population. The most common site of infection was cutaneous (27%), followed by isolated sinusitis (21%) and pulmonary and rhino-orbital-cerebral mucormycosis (each 18%). The most common isolated species were *Rhizopus* (50%) and *Mucor* (15%). Most patients received medical therapy with amphotericin B (79%), and more than half were treated surgically. The 1-year mortality rate reached 48%, with higher mortality observed in disseminated and rhino-orbital-cerebral infections than in other sites.

**Conclusion:**

Our study addressed the epidemiology of mucormycosis in Saudi Arabia and showed comparable patterns of clinical and mycological aspects to worldwide reports. Further studies are needed to evaluate mucormycosis risk factors and prognosis based on the species, site of infection and therapy type.

## Highlights

• First multi-center study in Saudi Arabia on clinical mucormycosis epidemiology

## 1 Introduction

The Mucorales are fungi that cause human disease; they are found worldwide in soil, decaying organic matter and contaminated foods. They were previously classified under the phylum of Zygomycetes, and infection with these agents was referred to as zygomycosis. (Ribes; Vanover-Sams; Baker, 2000). However, recent molecular phylogenies do not support the monophyly of the phylum, and the term zygomycetes has been abandoned. (Spatafora; Chang; Benny; Lazarus et al., 2016)

Mucorales commonly infect immunocompromised patients with a high fatality rate (Ribes; Vanover-Sams; Baker, 2000). Global incidence has increased in recent years, as the number of patients with predisposing factors has also risen significantly. Diabetes mellitus, metabolic acidosis and immunodeficiency states have been described as risk factors for acquiring mucormycosis infection (Prakash; Chakrabarti, 2019). An increase of mucormycosis infection has been evident in hematopoietic stem cell transplant recipients and patients with hematological malignancies (Kontoyiannis; Lionakis; Lewis; Chamilos et al., 2005; Lanternier; Sun; Ribaud; Singh et al., 2012; Marr; Carter; Crippa; Wald et al., 2002). The infection has been associated with natural disasters (Neblett Fanfair; Benedict; Bos; Bennett et al., 2012) and, most recently, with SARS-CoV-2 infection. The association with SARS-CoV-2 has mainly been observed in India, a country with a high prevalence of mucormycosis. The reason behind this phenomenon is still not fully understood; however, in a systematic review of patients with SARS-CoV-2 and mucormycosis co-infection, diabetes and steroid use were risk factors, reflecting that the immune status of the host plays a role in susceptibility to mucormycosis (Hoenigl; Seidel; Carvalho; Rudramurthy et al., 2022). Studies suggest that the epidemiology of mucormycosis is markedly different between countries (Prakash; Chakrabarti, 2019). The incidence and epidemiology of mucormycosis have not been thoroughly addressed in Saudi Arabia, with only sporadic reports of cases. Most cases reported from Saudi Arabia describe cutaneous zygomycosis (Al barrag et al., 2009; Al-Hedaithy, 1998; Al-Zaydani; Al-Hakami; Joseph; Kassem et al., 2015). There are a few other case reports of invasive mucormycosis with unfavorable outcomes (Al-Otaibi; Al-Shahrani; Al-Idrissi; Al-Abdely, 2016; Waness et al., Jul 2009) . A more recent study of 18 cases over 8 years reported mainly cutaneous and rhino-orbital-cerebral mucormycosis, with trauma being the predisposing risk factor and *Apophysomyces* the most commonly identified species (Elzein; Albarrag; Kalam; Arafah et al., 2020).

Due to the diversity in the clinical spectrum and mycology of mucormycosis worldwide, more data are needed to guide our understanding of the local epidemiology in Saudi Arabia. Therefore, our study aims to review the demographics, clinical manifestations and outcome of this infection in 3 tertiary care centers where mucormycosis cases are usually seen and treated.

## 2 Materials and Methods

### 2.1 Study design and setting

We conducted a retrospective multicenter study in 3 tertiary care centers in Saudi Arabia (King Abdulaziz Medical City-Riyadh, King Faisal Specialist Hospital and Research Center-Riyadh and King Abdullah Medical City-Makkah) from January 2009 to December 2019. All centers provide care for a large patient population, including solid organ and hematological malignancy patients, hematopoietic stem cell transplant recipients and solid organ transplant recipients.

### 2.2 Study population

Our study population included all patients with histopathologic or cytopathologic evidence of hyphae morphologically consistent with zygomycetes and associated tissue damage or patients with clinical and/or radiological evidence of infection and recovery of Mucorales by specimen culture obtained by a sterile procedure from a normally sterile site (Donnelly; Chen; Kauffman; Steinbach et al., 2020). Patient records were obtained from the hospitals' microbiology and histopathology databases. The following parameters were reviewed: demographics, risk factors, site of infection, microbiology, treatment modality and outcome.

### 2.3 Statistical analysis

Continuous data were described using median averages and interquartile ranges, while categorical data were described using frequencies and percentages. Data were collected using Microsoft Excel and entered into Statistical Package for the Social Sciences (SPSS) software for analysis.

## 3 Results

### 3.1 Demographic characteristics

A total of 33 patients were identified during the study period. The mean age was 42 years. People with diabetes accounted for 48% of the patient population, and 42.42% were diagnosed with hematologic malignancy. Solid organ and bone marrow transplant recipients represented a small proportion of the study population. No patients received deferoxamine therapy, were intravenous drug users, or were diagnosed with HIV. Further details are provided in Table 1.

**Table 1:** Baseline characteristics.

Patient characteristics	N= 33 (%)
Age (mean)	**42.23**
Males	20 (60.61)
Diabetes mellitus	16 (48.48)
Hematologic malignancy	14 (42.42)
Solid organ transplant	4 (12.12)
Liver	1
Renal	3
Bone marrow transplant	4 (12.12)
Renal failure	6 (18.18)
Liver cirrhosis	2 (6.06)
Prior antifungal therapy	10 (30.30)
Amphotericin B	1
Caspofungin	7
Voriconazole	2
Microbiological diagnosis	26 (78.79)
Site of infection	
Cutaneous	9 (27.27)
Isolated Sinusitis	7 (21.21)
Pulmonary	6 (18.18)
Rhino-orbital-cerebral	6 (18.18)
Gastrointestinal	2 (6.06)
Renal	1 (3.03)
Bone	1 (3.03)
Disseminated	1 (3.03)

### 3.2 Site of infection

The most common site of infection was cutaneous (27.27%), followed by localized sinusitis (21.21%) and pulmonary and rhino-orbital-cerebral mucormycosis, each representing 18.18% of cases (Table 1). Most patients with cutaneous, rhino-orbital and gastrointestinal mucormycosis also had diabetes, while infection of the sinuses and pulmonary system was commonly identified among patients with hematologic malignancies (Table 2).

**Table 2:** Underlying condition, microbiology, treatment and outcome of different sites of infection.

	Cutaneous	Sinus	Pulmonary	Rhino-orbital-cerebral	Gastrointestinal
**Underlying condition**					
DM	**4/9 (44.44)**§	3/7 (42.86)	2/6 (33.33)*	**4/6 (66.67)**	2/2 (100)
HM	1/9 (11.11)	**5/7 (**71.43**)**^	**4/6 (66.67)**	**4/6 (66.67)**#	-
HSCT	-	1/7 (14.29)	1/6 (16.67)	1/6 (16.67)	-
SOT	1/9 (11.11)	-	-	-	-
None	2/9 (22.22)	-	1/6 (16.67)	-	**-**
**Pathogen**					
*Rhizopus species*	**4/8 (50)**	**3/7 (42.86)**	-	**4/5 (80)**	1/2 (50)
*Mucor species*	**-**	2/7 (28.57)	**2/6 (33.33)**	-	-
*Rhizomucor species*	2/8 (25)	**-**	1/6 (16.67)	-	-
*Cunninghamella species*	2/8 (25)	**-**	-	-	-
*Lichtheimia corymbifera*	**-**	**-**	**2/6 (33.33)**	1/5 (20)	-
*Syncephalastrum species*	**-**	**-**	1/6 (16.67)	-	-
**Treatment**					
Surgical Therapy alone	1/9 (11.11)	-	-	-	-
Combination Surgical and Medical Therapy	**4/9 (44.44)**	**6/7 (85.71)**	2/6 (33.33)	**5/6 (83.33)**	-
Medical therapy	2/9 (22.22)	1/7 (14.29)	**3/6 (50)**	1/6 (16.67)	**2/2 (100)**
No Treatment	2/9 (22.22)	-	1/6 (16.67)	-	-
**Mortality**	3/9 (33.33)	4/7 (57.14)	3/6 (50)	4/6 (66.67)	1/2 (50)

DM diabetes mellitus, HM hematologic malignancy, HSCT hematopoietic stem cell transplant, SOT solid organ transplant

§ numbers in parenthesis represent percentages

⁎ both diabetic patients had hematological malignancy

# two patients with hematologic malignancy were diabetic

^ one patient had both diabetes and hematological malignancy

### 3.3 Mycology

**Figure 1:**
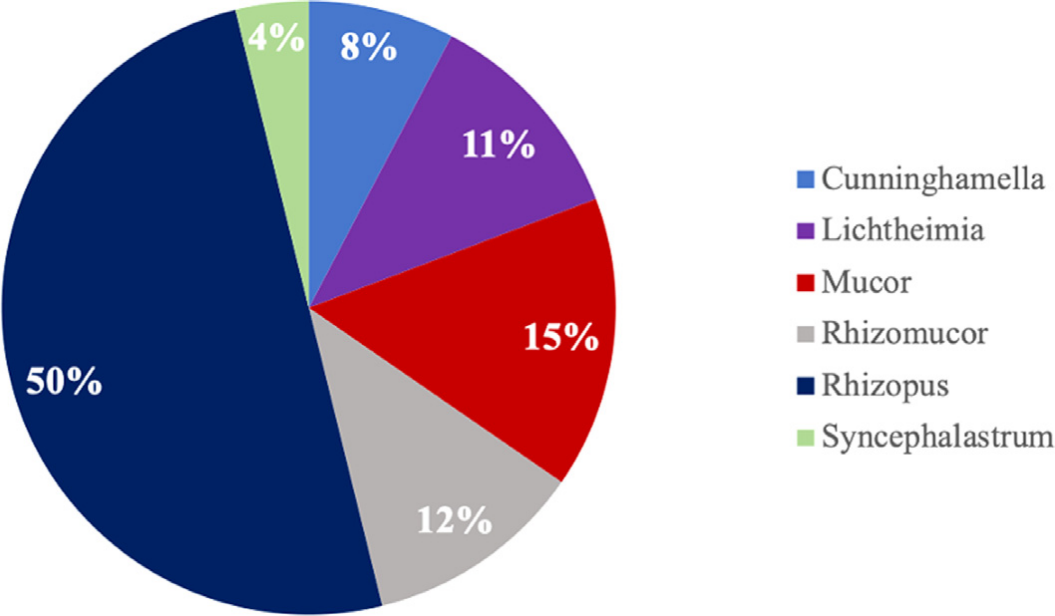
Isolated Mucorales Species (colored).

Cultures were positive in 26 cases ( 78.78% of the patient population), with *Rhizopus* species accounting for the majority of isolated organisms (50%), followed by *Mucor* species (15%). (Figure 1). *Rhizopus* was most the common species in all sites of infection except pulmonary mucormycosis, where *Lichtheimia corymbifera* and *Mucor* species were the most commonly identified organisms (Table 2).

### 3.4 Treatment

Therapy included the combination of surgical debridement and antifungal therapy in 18 of the 33 cases (54.54%), while 11 patients were treated with antifungal therapy alone (33.3%). Three patients did not receive any therapy; 2 had cutaneous involvement, and 1 was an immunocompetent patient with pulmonary involvement who refused treatment. One patient with cutaneous mucormycosis was treated with surgical therapy alone; medical therapy was not provided as the diagnosis was established post-mortem (Table 3).

**Table 3:** Mortality at one year by therapy and site of infection.

Treatment	N=33	Death/N (%)
Surgical therapy alone	1	**1/1(100)**
		Cutaneous 1/1
Antifungal and surgical therapy	18	**10/18 (55.56)**
		Sinuses 3/6
		Rhino-orbital-cerebral 3/5
		Cutaneous 2/4
		Pulmonary 2/2
		Renal 0/1
Antifungal therapy alone	11	**5/11 (45.45)**
		Gastrointestinal 1/2
		Pulmonary 1/1
		Sinuses 1/1
		Disseminated 1/1
		Rhino-orbital-cerebral 1/1
		Bone 0/1
		Cutaneous 0/4
No therapy	3	**0/3 (0)**
		Cutaneous 0/2
		Pulmonary 0/1

The most commonly used antifungal was amphotericin B (used in 26 of 33 patients, 78.78%), with amphotericin B lipid complex used in 15 of those cases and liposomal amphotericin B in the other 11 cases. Other agents used included Posaconazole in 2 patients and itraconazole in 1 patient.

Most patients with rhino-orbital-cerebral mucormycosis received surgical therapy (5 of 6 cases). Of patients with isolated sinusitis, 6 of 7 were also treated with surgical debridement; 1 patient with hematological malignancy was not treated surgically due to advanced disease and risk of complications. More than half of patients with cutaneous infection were treated surgically (5 of 9 cases). All patients with gastrointestinal mucormycosis were treated medically (Table 3). Granulocyte Colony Stimulating Factor was given to 5 of 33 patients; all were patients with hematologic malignancy and neutropenia and had sinusitis or rhino-orbital-cerebral involvement.

### 3.5 Mortality

Death within 1 year of diagnosis occurred in 16 patients (48.48%). Ten of 18 patients who received combined surgical and antifungal therapy died (55.56%), while 5 of 11 patients treated with antifungal therapy alone died (45.45%). Patients with rhino-orbital-cerebral infection had the highest mortality among infection sites, with 4 deaths occurring in the 6 cases. Pulmonary and isolated sinusitis mortality occurred in 3 of 6 and 4 of 7 cases, respectively. Three of 9 patients with cutaneous infection died; 1 had hematologic malignancy, 1 was a liver transplant recipient, and 1 had diabetes and extensive cutaneous involvement treated with surgical debridement only as the diagnosis was established post-mortem. One patient with disseminated infection died, and another with gastrointestinal infection who was too critically ill for surgical therapy and was treated with amphotericin B only (Table 3).

## 4 Discussion

Our study has illustrated the baseline clinical characteristics, presentation, mycology and outcome of mucormycosis in Saudi Arabia. The observed young, predominantly male patient population is similar to a global review of more than 900 reported cases (Roden; Zaoutis; Buchanan; Knudsen et al., 2005). Diabetes is the most common risk factor in our population, similar to worldwide reports; it remains the most commonly identified risk factor, even with the emergence of SARS-CoV-2-associated mucormycosis (Bhanuprasad; Manesh; Devasagayam; Varghese et al., 2021; Chakrabarti; Das; Mandal; Shivaprakash et al., 2006). Our patient population had a similar percentage of patients with malignancy and recipients of solid organ and stem cell transplants to reported cases worldwide (Jeong; Keighley; Wolfe; Lee et al., 2019). The incidence in this specific subpopulation has also been observed in regional reports from the Middle East (Stemler; Hamed; Salmanton-García; Rezaei-Matehkolaei et al., 2020). By contrast, in a study conducted by Alzein et al. in Saudi Arabia, trauma was the leading risk factor identified in the patient population; this was attributed to the fact that the hospital under study was a trauma center and the majority of patients were immunocompetent (Elzein; Albarrag; Kalam; Arafah et al., 2020). Those results were not reflected in our study, likely due to the different patient population treated in the centers in our study.

Worldwide, including in the Middle East, rhino-orbital-cerebral mucormycosis is the most commonly reported site of infection (Jeong; Keighley; Wolfe; Lee et al., 2019; Stemler; Hamed; Salmanton-García; Rezaei-Matehkolaei et al., 2020). However, in our population, the most common site of infection was cutaneous. Despite the similarity of site infection in our study and the report from Alzein et al. (Elzein; Albarrag; Kalam; Arafah et al., 2020), our patients with cutaneous mucormycosis were mainly immunocompromised with no apparent major traumatic injury. Isolated sinusitis involvement in our study was similar to regional reports (Stemler; Hamed; Salmanton-García; Rezaei-Matehkolaei et al., 2020). Pulmonary involvement was common among our patient population, likely due to the immunocompromised targeted population with patients with hematological malignancy representing a higher proportion compared with other local studies and studies in India (Chakrabarti; Das; Mandal; Shivaprakash et al., 2006). Hematological malignancy predominated in patients with pulmonary infection and isolated sinusitis consistent with the global epidemiology (Prakash; Chakrabarti, 2019).

The most commonly identified species was *Rhizopus* species, consistent with the global epidemiology (Prakash; Chakrabarti, 2019). *Mucor, Lichtheimia* and *Rhizomucor* species were the next most commonly isolated, which differs from the available worldwide (Roden; Zaoutis; Buchanan; Knudsen et al., 2005), regional (Stemler; Hamed; Salmanton-García; Rezaei-Matehkolaei et al., 2020) and local data (Elzein; Albarrag; Kalam; Arafah et al., 2020).

*Rhizopus* species accounted for the majority of isolates from patients with rhino-orbital-cerebral involvement, which is similar to reported studies; however, it was also the most commonly identified species in cutaneous forms in contrast to previous reports where *Apophysomyces* is the most commonly isolated species (Elzein; Albarrag; Kalam; Arafah et al., 2020; Prakash; Ghosh; Rudramurthy; Singh et al., 2019). In our study, *Lichtheimia* and *Mucor* were the most commonly identified species in pulmonary mucormycosis differing from reported cases around the world where *Cunninghamella* was more commonly reported in pulmonary disease (Jeong; Keighley; Wolfe; Lee et al., 2019).

Treatment of mucormycosis remains challenging. The current global guidelines recommend a combination of surgical intervention and antifungal therapy with high dose liposomal amphotericin B as the first line agent (Cornely; Alastruey-Izquierdo; Arenz; Chen et al., 2019). Most of our study population received amphotericin B; however, only half were treated with surgical debridement due to the high risk of surgery and/or patient preference. The overall mortality among our population was approximately 50%, comparable with worldwide cases; the highest mortality was observed in cases with rhino-orbital-cerebral involvement (Jeong; Keighley; Wolfe; Lee et al., 2019). Patients treated surgically and with antifungal therapy had higher mortality than those treated with antifungal therapy alone; this was likely related to the fact that patients treated surgically more commonly had rhino-orbital-cerebral involvement.

The retrospective nature of our study limited the accuracy of assessing clinical outcome apart from mortality. The small sample size was also a limitation; this was due to the low prevalence of the infection. However, ours is the largest study of mucormycosis in Saudi Arabia and should help build an idea of local epidemiology.

Ours is the first multi-center study addressing the epidemiology of mucormycosis in Saudi Arabia. It illustrates the variable clinical and mycological aspects of mucormycosis, showing findings comparable to international reports. Further prospective advanced epidemiological studies in different regions of the kingdom are needed to better reflect the prevalence of mucormycosis. Comprehensive data on individual presentation and Mucorales species are required to inform the best approach to managing mucormycosis.

## Ethical Approval Statement

The authors confirm that the ethical policies of the journal, as noted on the journal's author guidelines page, have been adhered to and the appropriate ethical review committee approval received. The study was approved by King Abdullah international medical research center in Riyadh (RC19/334/R) and approved by the institutional review board in each participating center.

## Funding

This research did not receive any specific grant from funding agencies in the public, commercial or not-for-profit sectors.

## Declaration of Competing Interest

The authors have no conflict of interest to disclose.
